# Supplementary material for: A multi-dimensional incomplete stepped-wedge trial design to estimate the impact of standards-based audit
Source: PLoS One. 2023 Nov 30;18(11):e0294352. doi: 10.1371/journal.pone.0294352 (PMC10688679; doi:10.1371/journal.pone.0294352)
Supplement: Attachment — Submitted filename: Comments-1.docx [file pone.0294352.s001.docx]

This study is highly intriguing as it employs a complex research design to investigate the impact of standards-based audits. The design incorporates multiple dimensions and utilizes a stepped-wedge trial approach. In this approach, the application of the intervention (standards-based audits) varies across different clusters, rather than being uniform. The primary objective is to estimate the effects of these audits on specific outcomes.

The study introduces an innovative trial design that avoids the common pitfalls associated with the use of before-after designs in clinical audits, which often lead to biased impact estimations. However, there are certain aspects that, in my opinion, the authors should carefully contemplate before proceeding with the publication of their work in your journal.

Neither in the introduction nor in the discussion sections did the authors clearly explain why they believe the chosen design would be more advantageous when compared to existing approaches (such as before and after designs). I would appreciate it if the authors could offer readers a comprehensive justification for the trial design and elaborate on its distinctions from the before-after design used in clinical audits. What limitations of the before-after design have influenced their preference for the trial design?

In the body text, I recommend placing tables immediately after their corresponding citations. This approach not only adheres to the journal's guidelines but also enhances the reader's comprehension flow.

I am of the opinion that there are certain writing issues that should be addressed on a line-by-line basis. For instance, in line number 115 of the manuscript, the in-text citation format appears inconsistent with other citations, and information source for Table 1 is labeled as "source:2".

Overall, this is a commendable piece of work with significant novelty, and it deserves support. If published, it has the potential to benefit numerous researchers and contribute to the ongoing improvement of our audit practices, particularly in developing countries.
