# Supplementary material for: A multi-dimensional incomplete stepped-wedge trial design to estimate the impact of standards-based audit
Source: PLoS One. 2023 Nov 30;18(11):e0294352. doi: 10.1371/journal.pone.0294352 (PMC10688679; doi:10.1371/journal.pone.0294352)
Supplement: Attachment — Submitted filename: Response_To_Reviewers_18Sep23.docx [file pone.0294352.s002.docx]

Liverpool School of Tropical Medicine

Pembroke Place

Liverpool

L3 5QA

United Kingdom

18^th^ September 2023

To:	The editor, PLOS ONE

Dear Editor,

Thank you for the helpful feedback from the reviewers concerning our manuscript. I am now submitting a revised manuscript entitled “*A multi-dimensional incomplete stepped-wedge trial design to estimate the impact of standards-based audit”* by Florence Mgawadere and me.

Below are our responses to each of the reviewers’ and editors' comments.

| Reviewer’s comment | Response |
| --- | --- |
| Well written and fairly explained. Just I would like to know more about considerations after those limitation points. For example, you have mentioned in line 502 (the covariate maybe considered) and that (in my opinion) will let the reader be thrilled and imagining what will these improvements do next? | Thank you for your interest in our manuscript and your desire to know more. The point noted is a comment regarding a possible approach to the modelling of a particular circumstance. Our aim in the manuscript is to present the design and the potential value of using the design.  In the example noted we suggest a possible approach to handling a particular circumstance; the potential value of the approach would need to be explored in the context of a particular study design. We have not amended the text in response. |
| Neither in the introduction nor in the discussion sections did the authors clearly explain why they believe the chosen design would be more advantageous when compared to existing approaches (such as before and after designs). I would appreciate it if the authors could offer readers a comprehensive justification for the trial design and elaborate on its distinctions from the before-after design used in clinical audits. | Thank you for highlighting the need to provide justification for the design and distinguish it from the before-after design. Text has been added as follows:  Lines 105-110 indicate the ways in which the SW-CRT design is related to the before-after design but also provides a mechanism for addressing any bias in estimation due to a secular trend, which the before-after design cannot do.  Lines 152-3 have been added to clarify an aspect of the extension that should be mentioned. |
| What limitations of the before-after design have influenced their preference for the trial design? | Sentences have been added in lines 55-57 to indicate the limitations and line 87 has been modified. |
| In the body text, I recommend placing tables immediately after their corresponding citations. This approach not only adheres to the journal's guidelines but also enhances the reader's comprehension flow. | The guidelines indicate that each table should be placed immediately after the paragraph in which the first citation of a table occurs. I have verified that this is how they are positioned (and inserted some page breaks to avoid tables and Figures being split across pages). |
| I am of the opinion that there are certain writing issues that should be addressed on a line-by-line basis. For instance, in line number 115 of the manuscript, the in-text citation format appears inconsistent with other citations, and information source for Table 1 is labeled as "source:2". | Thank you for identifying these issues which have now been addressed (line 115 is now line 121).  Table 1 has been revised to acknowledge the content from the reference cited more accurately in the format used in Table 2 (as no comment was raised for Table 2 I presume it is correct; I can’t find guidance on this in the webpages). |
| Overall, this is a commendable piece of work with significant novelty, and it deserves support. If published, it has the potential to benefit numerous researchers and contribute to the ongoing improvement of our audit practices, particularly in developing countries. | Thank you for your endorsement of the value of this work. |
| Please ensure that your manuscript meets PLOS ONE's style requirements, including those for file naming. | These have now been checked, and Figures no longer contain legends etc |
| Please review your reference list to ensure that it is complete and correct. If you have cited papers that have been retracted, please include the rationale for doing so in the manuscript text, or remove these references and replace them with relevant current references. | All references were checked on 4^th^ Sept 2023.  Some details have been revised to ensure compliance with standards for references.  The reference we numbered 1 is no longer available directly via the WHO webpages. We have therefore referenced an alternative (previously numbered 6). This change has necessitated some revision of reference numbers (one of which was identified to have been incorrect). |

Yours sincerely,

Sarah White
